# Medical News and Items

**Published:** 1877-07

**Authors:** 


### Medical News and Items.

SALE *vs.* THE LOUISVILLE MEDICAL COLLEGE.—Our readers, who have noticed a report of this suit in the June number of this periodical, may also hear "*alteram partem.*" Here is their account, as published over E. S. Galliard's signature, in the Richmond and Louisville *Medical Journal*:

"In September last, Mr. Sale, a medical student and the plaintiff in this suit, entered the college mentioned. He paid his fees. A few weeks subsequently he was offered (with others) free tuition in a Louisville Medical Institution. This offer was a part of the sworn testimony of the plaintiff. He accepted it and requested a return of his money. This request was of course not granted. He then asked for his tickets. He was told that this College never gave its tickets (the evidence of attendance upon a course of lectures) until the last month of the course. Had the tickets been given there would have been no suit; but they were withheld, and the suit invited. The plea in this suit was failure to comply with promises made. In the garbled version of the magistrate's decision published in the "*Courier-Journal*," and sent to the Medical Press everywhere, and to the alumni of the Louisville Medical College, it is admitted that this plea could not be, and had not been, sustained by the evidence. The so-called "judgment" of the magistrate was given on the ground that the present Faculty were not legally elected.

"The Book of Minutes of the Proceedings of the Board of Trustees shows that the members of the Faculty were not only legally elected by the Present Board, but that one of the last acts of the old Board was to elect them (with one exception) before adjournment. It may be asked why was not this book produced and such a "judgment" prevented. The answer is simple; it was in possession of the persecuted Secretary of the Board of Trustees, Dr. B. M. Wible, who was ill and soon after died, and was found after his death, and after the socalled "judgment" had been "rendered," and copies of it forced into a daily paper (which never publishes the petty business of a magistrate's court), and actively disseminated for purposes too evident to require indication."

AT A MEETING of the Board of Regents, Kentucky Infirmary for Women and Children, held May 15, 1877, the following persons were elected to serve for the ensuing year:

MEDICAL STAFF.

Dr. David Cummins, deformities and general surgery; Dr. Geo. W. Griffiths, diseases peculiar to women and children; Dr. Richard C. Brandeis, diseases eye, ear and throat; Dr. Ely McClellan, pelvic surgery; Dr. Samuel Brandeis, surgeon accoucheur; Dr. James McEvoy, clinical assistant.

DENTAL STAFF.

B. Oscar Doyle, D. D. S.; W. T. Redman, D. D. S., M. D.; Ch. E. Dunn, D. D. S.; W. T. Goddard, D. D. S.; C. G. Edwards, D. D. S.

ADDITIONAL HONORARY MEMBERS BOARD REGENTS.

Prof. W. H. Byford, Chicago, Ill.; Dr. Washington Atlee, Philadelphia, Pa.; Dr. J. Marion Sims, New York.

THE FIRST ANNUAL MEETING of the American Dermatological Association will be held at Niagara Falls on the fourth day of September next. The titles of all papers to be read at any annual session shall be forwarded to the Secretary, not later than one month before the first day of the session.

MORTALITY IN THE CITY OF CHICAGO DURING THE FIVE WEEKS, from May 19 to June 23, 1877. Total number of deaths, 620; males, 343; females, 277; under one year, 164. Principal causes of death: accidents, 22; apoplexy, 14; bronchitis, 12; convulsions, 68; diphtheria, 19; enteritis, 19; cholera infantum, 8; diseases of heart, 9; kidneys, 2; pleurisy, 4; pneumonia, 26; phthisis, 63; measles, 10; scarlet fever, 96; typhoid fever, 9; meningitis, 22.

